# Lower Limb Kinematics of People With Midfoot Osteoarthritis During Level Walking and Stair Climbing

**DOI:** 10.1002/jfa2.70054

**Published:** 2025-06-09

**Authors:** Merridy J. Lithgow, Jayishni N. Maharaj, Andrew K. Buldt, Shannon E. Munteanu, Benjamin F. Mentiplay, Hylton B. Menz

**Affiliations:** ^1^ Discipline of Podiatry School of Allied Health Human Services and Sport La Trobe University Victoria Australia; ^2^ School of Health Sciences and Social Work Griffith University Gold Coast Australia; ^3^ Discipline of Sport and Exercise Science School of Allied Health Human Services and Sport La Trobe University Victoria Australia

**Keywords:** biomechanical phenomena, foot, foot joints, gait, midfoot, osteoarthritis

## Abstract

**Background:**

Midfoot osteoarthritis (OA) affects one in eight people over 50, yet its impact on foot and lower limb kinematics remains poorly understood. This study compared foot and lower limb kinematics during level walking and stair climbing between people with and without symptomatic radiographic midfoot OA.

**Methods:**

Symptomatic radiographic midfoot OA was defined as midfoot pain in the last 4 weeks and radiographic OA in one or more midfoot joints. Cases aged ≥ 45 years were matched 1:1 for sex and age (± 5 years) to controls. A 10‐camera motion analysis system was used to capture foot and lower limb kinematics during level walking and stair climbing, which were analysed with a validated multi‐segmental lower limb model. Group differences were analysed using independent samples *t*‐tests and effect sizes for discrete angles, whereas statistical parametric mapping compared kinematic patterns between groups.

**Results:**

We included 24 midfoot OA cases (mean age 64.4, SD 9.5) matched to 24 controls (mean age 65.2, SD 10.1). During level walking, people with midfoot OA walked slower and displayed absolute joint angles that showed less hip extension throughout stance, less knee flexion in early and late stance, less ankle dorsiflexion throughout stance (medium to large effects), greater subtalar pronation in late stance, and greater tarsometatarsal supination during early stance (medium effects). There were few differences during stair ascent and descent.

**Conclusion:**

People with midfoot OA walk slower and demonstrate medium to large differences in sagittal plane hip, knee, and ankle kinematics, and medium differences in subtalar and tarsometatarsal kinematics. These findings offer insights into the walking patterns of people with midfoot OA and the mechanisms that may contribute to or result from the condition. Prospective studies are needed to clarify the temporal relationship between these factors and midfoot OA development.

Midfoot osteoarthritis (OA) is a common and disabling condition characterised by arthritic change in one or more joints of the midfoot [1], occurring in one in eight people aged greater than 50 years [2]. People with midfoot OA report greater foot pain and poorer foot function compared to those without the condition [3, 4]. Additionally, foot OA poses a significant burden on the healthcare system through its high prevalence, resource utilisation, impact on general practice workloads, and economic costs [5]. In contrast to the extensive research conducted on lower limb kinematics in OA affecting the hip [6], knee [7], and ankle [8], studies centred on midfoot OA are sparse [9], highlighting the necessity for further research in this area.

Previous research has demonstrated that people with midfoot OA have a characteristic foot posture and walk differently to those without [9, 10, 11]. Clinical and radiographic findings indicate that lower limb characteristics associated with midfoot OA include a more pronated foot posture, reduced range of motion at the subtalar joint and first metatarsophalangeal (MTP) joints, decreased foot and leg muscle strength, and increased first cuneiform‐metatarsal joint mobility [9, 11] compared to those without midfoot OA. They also display plantar pressures associated with lowering of the medial longitudinal arch, greater lateral push off, and less propulsion at toe‐off [12].

The lower limb kinematics of people with midfoot OA have been investigated in two studies [10, 13]. Rao et al. [13] found that people with midfoot OA exhibited less first metatarsal plantarflexion excursion during level walking, but greater calcaneal eversion during stair descent. The main limitation of this study was the use of a simple five‐marker foot model, which provided only five foot joint measurements. Deschamps et al. [10] examined the lower limb kinematics of people with both midfoot and subtalar joint OA and found sagittal plane ankle joint range of motion during the pre‐swing phase was smaller in those with OA relative to controls, whereas the sagittal plane tarsometatarsal joint range of motion in the pre‐swing phase was greater. The main limitation of Deschamps et al. [10] study was its small sample size (*n* = 10). Although this study employed a more sophisticated biomechanical model [14], it may not adequately represent the tri‐planar motion of the foot during gait [15].

Previous studies have also been limited with regard to how midfoot OA is defined, and the statistical analyses that have been used. Firstly, both Rao et al. [13] and Deschamps et al. [10] used the Kellgren and Lawrence [16] scale to diagnose midfoot OA, which has been criticised for relying on osteophyte presence and assuming a chronological sequence in OA development [17]. Secondly, a relatively new analytical approach called statistical parametric mapping (SPM) [18] has evolved in recent years and has not been used for the analysis of lower limb biomechanics in midfoot OA. The benefits of using SPM include its ability to analyse the entire gait cycle continuously rather than at discrete points. SPM offers greater sensitivity and can detect subtle differences that might be overlooked when only specific points in the gait cycle are examined [18, 19, 20].

Understanding the differences in foot and lower limb kinematics between people with and without midfoot OA provides insights into the effects of midfoot OA on kinematic gait patterns, such as changes in joint position. Walking during daily activities includes both level ground and stair ambulation. Given the potential mobility restrictions associated with midfoot OA, stair negotiation may reveal compensatory strategies not observed during level walking, similar to those observed in OA affecting other joints [21]. Using a reliable radiographic foot atlas [1] to determine the presence of midfoot OA, the objective of this study was to compare foot and lower limb kinematics between people with and without symptomatic radiographic midfoot OA, using a validated lower limb biomechanical model that incorporates tri‐planar movements within the foot [15]. It was hypothesised that people with midfoot OA would have altered foot and lower limb kinematics compared to those without the condition.

## 1 Methods

### 1.1 Study Design

Adults aged 45 years or older were invited to participate. Participants were recruited through social media (*n* = 29), university faculty or family (*n* = 11), participant referral (*n* = 7), local retirement village (*n* = 5), library flyers (*n* = 2), health practitioner (*n* = 1), and local orthopaedic hospital (*n* = 1). Ethical approval was acquired from the La Trobe University Human Research Ethics Committee (HEC19538). Written informed consent was obtained from all participants.

### 1.2 Midfoot OA Case Definition

Midfoot OA was diagnosed based on the presence of symptoms and radiographic features of OA in the midfoot. Weight‐bearing dorsoplantar and lateral radiographs were obtained. For the cases, foot pain location in the last 4 weeks was ascertained by shading a foot manikin (The University of Manchester, 2000. All rights reserved) [22]. Symptomatic, radiographic midfoot OA was defined as pain located in the midfoot region (≥ 3/10 visual analogue scale [VAS]) in the last 4 weeks [22, 23, 24], and a radiographic score of two or more for osteophytes or joint space narrowing on either weight‐bearing dorsoplantar or lateral views in one or more midfoot joints (first cuneometatarsal, second cuneometatarsal, navicular‐first cuneiform, and talonavicular) in the same foot using the La Trobe Foot Atlas [1]. For bilateral cases, the most painful foot was selected for testing. Symptomatic, radiographic midfoot OA cases were then sex‐ and age‐matched to controls with a 5‐year tolerance for age. Controls had no pain anywhere in the foot, and no radiographic midfoot OA according to the La Trobe Foot Atlas case definition. The right foot was chosen as the index foot for controls. For both cases and controls, we excluded those who reported (i) surgery to any of the musculoskeletal structures in the foot, (ii) a history of any fractures in the midfoot, and (iii) inflammatory joint conditions or neurological diseases.

### 1.3 Descriptive Characteristics of Participants

Descriptive characteristics were collected via a secure structured online questionnaire using REDCap (Research Electronic Data Capture, Vanderbilt University) [25, 26]. The Manchester‐Oxford Foot Questionnaire [27] and Short Form 12 [28] questionnaires were also obtained. Midfoot OA cases were provided with a foot manikin [22] and indicated any foot pain they had in the last month that lasted one day or longer. They also rated their current level of pain and stiffness in their midfoot joints using a visual analogue scale (scored from 0 [no pain] to 100 [worst pain imaginable]) [29].

### 1.4 Clinical Assessments

All clinical assessments were undertaken at the La Trobe University Foot and Ankle Laboratory. Height and weight were measured using a stadiometer and scales, and body mass index (BMI) was calculated. Static foot posture was assessed using the Foot Posture Index (FPI) [30]. Clinical measures of foot and ankle range of motion were assessed using the knee to wall test [31], first MTP joint maximum dorsiflexion [32], and active ankle joint inversion/eversion [33].

### 1.5 Biomechanical Data Collection

Biomechanical data were collected at the La Trobe University Gait Laboratory. Reflective markers were placed on the foot (9 mm) and on the pelvis, thigh, leg and foot (14 mm) [15, 34]. The marker set consisted of a four‐marker cluster on the thigh and shank segments and anatomical markers placed over the anterior‐superior iliac spines, posterior superior iliac spines, medial and lateral femoral epicondyles, and medial and lateral malleoli. For the feet, a five‐segment marker set was used to track the motion of four joints: the ankle, subtalar, midtarsal, tarsometatarsal and MTP joints [15, 34]. This model, which combined two marker sets, allowed us to measure the motion of the hip, knee, ankle, and foot joints.

A 10‐camera opto‐reflective motion capture system (Vicon Motion Systems Pty Ltd. Oxford, UK) recorded marker trajectories at a sampling rate of 200 Hz. Ground reaction force (GRF) data were recorded at a sampling rate of 1000 Hz using two small (600 mm × 400 mm) force plates (Advanced Mechanical Technology Inc. Watertown, MA, USA) embedded in the laboratory floor. Marker trajectories and GRF data were simultaneously recorded using Vicon Nexus software (Vicon Motion Systems Pty Ltd, Oxford, UK). A static trial was first captured, and then participants completed overground walking, followed by stair climbing (ascent and descent). For walking, there was a short acclimatisation period, then participants were instructed to walk at a comfortable self‐selected speed along a 14‐m walkway until at least three successful trials were obtained. A successful trial was defined whereby walking speed and ground contact time was within 10% of the participant's average, and a whole foot contact occurred on the force plate of the index foot. The biomechanical data collection method for stair climbing is detailed in Supporting Information S1.

### 1.6 Musculoskeletal Modelling

**FIGURE 1:**
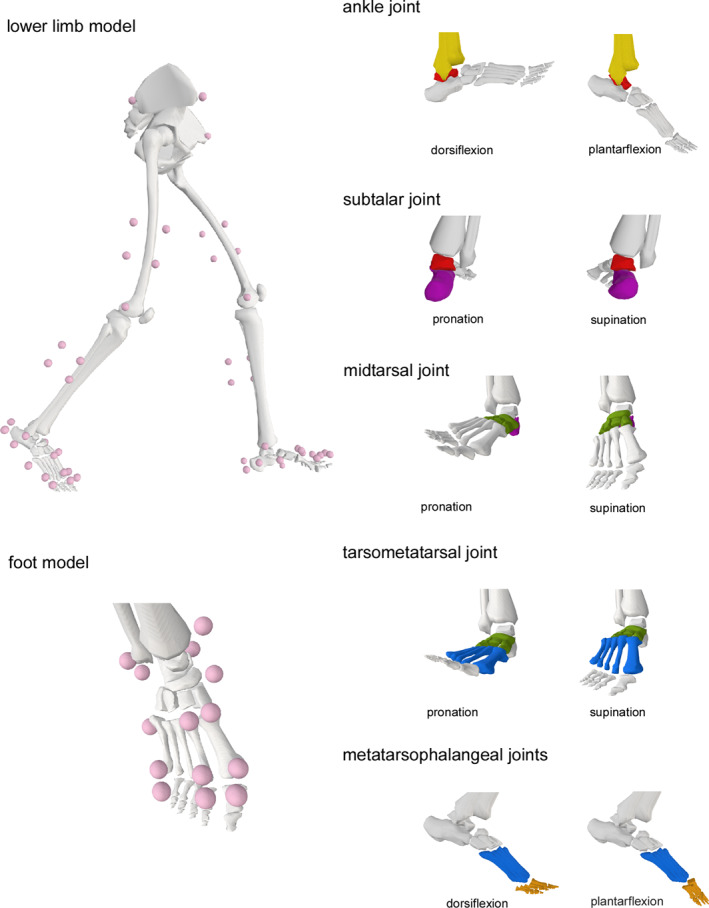
Lower limb model and depiction of foot movements.

A validated multi‐segment foot model embedded in a lower limb model was implemented in OpenSim software [35] for kinematic analysis. The model is capable of measuring motion across four joint complexes: the subtalar joint, the midtarsal joint, the tarsometatarsal joint, and MTP joints 1–5. The model facilitates tri‐planar motion, particularly enabling pronation and supination at the subtalar, midtarsal, and tarsometatarsal joints, along with dorsiflexion and plantarflexion at the MTP joints. The details of the musculoskeletal modelling and marker location have been published previously (see Supporting Information S2) [15, 34]. Figure 1 shows the lower limb model and foot movements.

### 1.7 Biomechanical Data Processing

Initial contact and toe‐off were used to define the start and finish, respectively, of stance phase. For walking, initial contact and toe‐off were defined as the first frame where the raw vertical ground reaction force data exceeded or dropped below 20 N, respectively. For stair climbing, initial contact and toe‐off were determined through visual inspection of the marker trajectories.

Raw marker positions were filtered in Matlab (Mathworks, R2023a, Natrick, MA) using a zero‐lag, second‐order, low‐pass Butterworth filter with a cutoff frequency of 25 Hz, prior to performing inverse kinematics analyses through OpenSim [35, 36]. Joint angle outputs were subsequently filtered using a bi‐directional, second‐order low‐pass Butterworth filter with a cut‐off frequency of 10 Hz.

The kinematic variables of interest were sagittal plane angles of the hip, knee, ankle and MTP joints 1–5, and tri‐planar movement of the subtalar, midtarsal and tarsometatarsal joints. Processed kinematic data were time‐normalised to 101 points by linear interpolation from heel strike to toe‐off and averaged over three trials.

Key spatiotemporal variables were walking velocity and absolute ground contact time during stance (initial contact to toe‐off). Variables were calculated as the mean of three trials. If any trial deviated in time to complete the task by more than 10% for level walking and 15% for stairs it was excluded, and an alternative trial within the threshold was selected.

### 1.8 Statistical Analysis

Data were analysed using IBM SPSS Statistics version 29.0 (IBM Corp, Armonk, NY, USA). To compare cases and controls for the participant characteristics and clinical assessments, continuous variables were analysed with independent samples *t*‐tests, while categorical variables were examined using chi‐square (χ^2^) tests. Independent samples *t*‐tests were performed to assess for differences in walking velocity and ground contact times between cases and controls. No imputation for missing data was undertaken. A formal a priori sample size calculation was not undertaken given the lack of existing data. However, using G*Power [37], based on a two‐tailed independent samples *t*‐test, our sample size of 24 participants per group was sufficient to detect differences in effect size of 0.83, with an error probability of 0.05% and 80% power. While this reflects a large effect size, it is consistent with previous research. For example, Deschamps et al. [10] investigated sagittal plane motion of the tarso‐metatarsal joint during midstance in people with midfoot OA and controls, each group comprising 10 participants. They reported mean values of 2.8 ± 2.0° for the midfoot OA group and 7.5 ± 1.6° for controls. These results demonstrated a statistically significant mean difference of 4.7° [95% CI: 2.9, 6.3], *p* = 0.002, corresponding to a moderate effect size (Cohen's *d* = 0.44). This supports the appropriateness of our sample size to detect differences of similar or larger magnitude.

Kinematic data were analysed using two approaches. Firstly, differences in absolute angles between the groups were compared according to the temporal events described by Perry and Burnfield [38] (heel contact [0%], end of loading [20%], end of midstance [50%], end of terminal stance [83%], and end of pre‐swing [100%]). Secondly, differences between groups were compared using 1‐dimensional SPM [18]. SPM was performed using the spm1D package (v. 0.4.8, https://spm1d.org/ [19]) in Matlab. For both methods, the main effects were tested using independent samples *t*‐tests. For the temporal events method, effect sizes for between‐group comparisons were calculated using Cohen's *d* and were interpreted as follows: *<* 0.1 = tiny, 0.1 to *<* 0.2 = very small, 0.2 to *<* 0.5 = small, 0.5 to *<* 0.8 = medium, 0.8 to *<* 1.2 = large, 1.2 to 2 = very large, d > 2 = huge [39]. A finding was considered important when the *p*‐value was < 0.05 and had at least a medium effect size. By combining both methods, we sought to identify overall trends throughout the entire gait cycle using SPM, while still focussing on critical moments during the gait cycle with discrete measures.

## 2 Results

### 2.1 Study Population

The sample consisted of 24 cases (mean age 64.4, SD 9.5, 22 females) and matched 1:1 for sex and age (± 5 years) to 24 controls (mean age 65.2, SD 10.1). The characteristics of participants are provided in Table 1. People with midfoot OA had a greater BMI and were more likely to take ≥ 4 medications compared to controls. They also reported lower physical health‐related quality of life (SF‐12) and demonstrated less ankle joint inversion and eversion range of motion.

**TABLE 1:** Descriptive characteristics of people with symptomatic midfoot OA and asymptomatic controls.

	Midfoot OA (*n* = 24)	No midfoot OA (*n* = 24)
Demographic characteristics		
Age, years	64.4 (9.5)	65.2 (10.1)
Sex, *n* (%) female	22 (91.7)	22 (91.7)
Body mass index, kg/m^2^ ^a^	32.1 (5.8)	25.9 (4.9)
Attended higher education, *n* (%)	12 (50)	16 (66.7)
Cost of living: can manage without much difficulty, *n* (%)	11 (45.8)	9 (37.5)
Smoking status, *n* (%)		
Current daily basis	1 (4.2)	2 (8.3)
Previous daily basis	8 (33.3)	5 (20.8)
Never smoked	13 (54.2)	16 (66.7)
Ethnicity, *n* (%)		
Oceanian	16 (66.7)	18 (75)
United Kingdom	4 (16.7)	1 (4.2)
South‐East Asian	1 (4.2)	4 (16.7)
Southern and Eastern European	2 (8.3)	0 (0)
North‐West European	1 (4.2)	1 (4.2)
Self‐reported medical conditions, *n* (%)		
Osteoarthritis^a^	18 (75.0)	4 (16.7)
Hypertension	9 (37.5)	6 (25)
Leg cramps	8 (33.3)	5 (20.8)
Hearing impairment	5 (20.8)	3 (12.5)
Hypercholesterolaemia	1 (4.2)	2 (8.3)
Diabetes	3 (12.5)	1 (4.2)
Heart disease	2 (8.3)	1 (4.2)
Fibromyalgia	2 (8.3)	0 (0)
Depression	3 (12.5)	0 (0)
Asthma	2 (8.3)	2 (8.3)
Osteoporosis	1 (4.2)	1 (4.2)
Thyroid condition	1 (4.2)	3 (12.5)
Self‐reported medications, *n* (%)		
Number of people who take ≥ 4 medications^b^	11 (45.8)	2 (8.3)
Foot pain and function		
Midfoot pain duration (weeks)	279.5 (268.0)	—
Midfoot pain severity (VAS)	63.0 (13.7)	—
Midfoot pain stiffness (VAS)	48.8 (26.8)	—
MOXFQ (for midfoot pain)	54.3 (20.4)	—
Health status		
SF‐12 (physical)^a^	39.2 (11.5)	54.4 (4.1)
SF‐12 (mental)	50.1 (11.0)	55.7 (6.8)
Clinical features		
FPI	4.8 (2.0)	3.9 (2.3)
AJ ROM ‐ knee to wall (°)	39.6 (7.3)	41.3 (7.8)
AJ inversion ROM (°)^b^	35.0 (6.0)	39.2 (5.7)
AJ eversion ROM (°)^a^	22.8 (4.9)	28.5 (4.9)
First MTP joint ROM (°)	72.4 (16.6)	77.9 (13.6)
Joint‐specific radiographic OA		
Total number of joints	2.1 (0.9)	—
First cuneometatarsal – *n* (%)	8 (33.3)	—
Second cuneometatarsal – *n* (%)	17 (70.8)	—
Navicular‐first cuneiform – *n* (%)	16 (66.7)	—
Talonavicular – *n* (%)	10 (41.7)	—

*Note:* Values are mean ± SD unless indicated otherwise. Midfoot pain severity was measured using a visual analogue scale (VAS) (0–100 mm), with higher scores indicating worse pain. Self‐reported medications, n = number of people taking specific class of drug.

Abbreviations: AJ: ankle joint; FPI: Foot Posture Index (supinated < 0, normal 0‐5, pronated > 6); HADS: Hospital Anxiety and Depression Scale; IPEQ: Incidental and Planned Activity Questionnaire. For the health status questionnaires, higher scores indicate higher levels of health outcomes; MOXFQ: The Manchester‐Oxford Foot Questionnaire; MTP: metatarsophalangeal; NSAIDs: Non‐steroidal anti‐inflammatory drugs; OA: osteoarthritis. Higher values indicate a greater range of movement. Joint‐specific OA does not tally to 100%, as ≥ 1 midfoot joint may have OA; ROM: range of movement; SF‐12: Short Form‐12.

^a^ *p* < 0.001.

^b^ *p* < 0.05.

### 2.2 Level Walking

**FIGURE 2:**
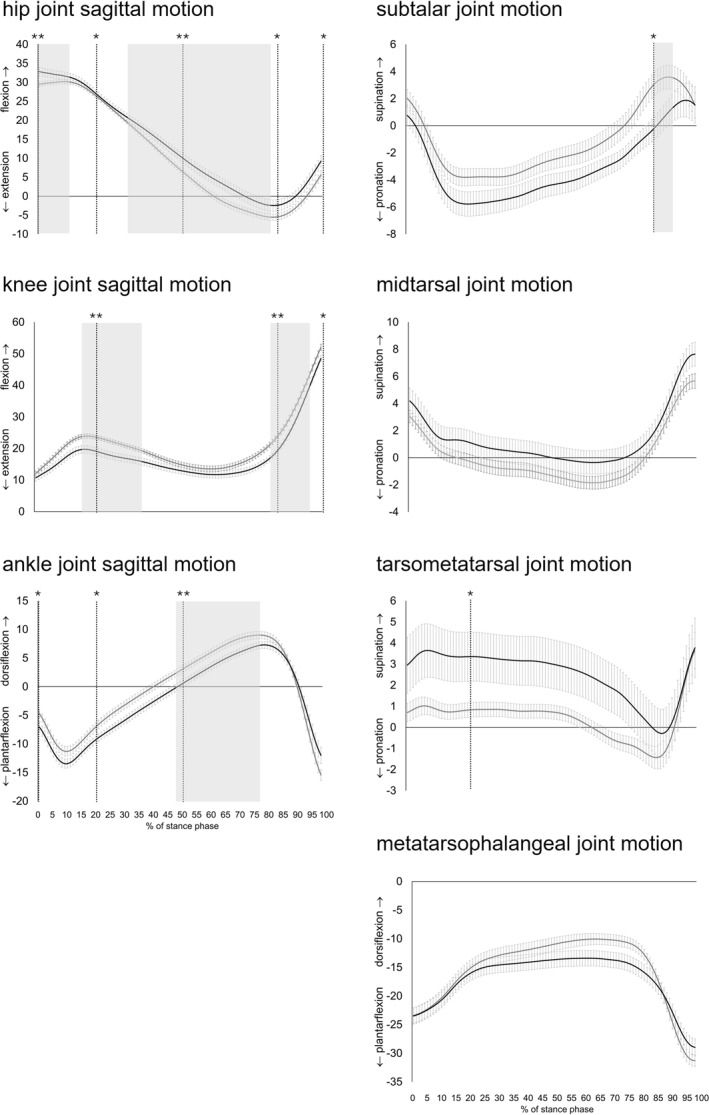
Absolute angles of lower limb joint kinematics during level walking in people with symptomatic midfoot OA and asymptomatic controls (mean and standard error bars). The black line represents the cases, and the grey line represents the controls. The horizontal axis shows the percentage of the stance phase, while the vertical axis indicates degrees of movement. Significant findings using the temporal events method are marked with vertical lines, with one asterisk representing *p* < 0.05 and two asterisks representing *p* < 0.01. Significant findings from the SPM analysis are highlighted with block shading.

People with midfoot OA walked slower (mean 0.97 m/s, SD 0.17) than controls (mean 1.21 m/s, SD 0.20; *p* < 0.001, *d* = 1.32: very large effect) and had greater ground contact times (mean 0.72, SD 0.09) than controls (mean 0.64 s, SD 0.05; *p* < 0.001, *d* = 1.12: very large effect). For the analysis of the absolute joint angles using the temporal events method, people with midfoot OA had greater hip flexion at heel contact, less hip extension at the end of loading, end of midstance and end of terminal stance, and greater hip flexion at the end of pre‐swing (see Table 2 and Figure 2). Additionally, they had less knee flexion at the end of loading, less knee flexion at the end of terminal stance and the end of pre‐swing, less ankle dorsiflexion at heel contact, the end of loading and end of midstance, greater subtalar pronation at the end of terminal stance and greater tarsometatarsal supination at the end of loading. Significant findings using the temporal events method are depicted as vertical lines in Figure 2.

**TABLE 2:** Absolute angles of lower limb joint kinematics during level walking in people with symptomatic midfoot OA and asymptomatic controls.

		Cases (*n* = 24)	Controls (*n* = 24)	Mean difference (95% CI)	*p*‐value	Effect size (Cohen's *d*)	Interpretation
Hip joint – sagittal	Heel contact (0%)	33.4 (5.8)	28.7 (2.9)	−4.7 (−7.5 to −1.9)	0.002	1.08	Large
	End of loading (20%)	27.7 (4.9)	25.2 (2.6)	−2.5 (−4.9 to −0.1)	0.042	0.67	Medium
	End of midstance (50%)	11.2 (6.1)	5.8 (3.7)	−5.4 (−8.5 to −2.3)	< 0.001	1.10	Large
	End of terminal stance (83%)	−1.9 (7.4)	−6.5 (4.2)	−4.6 (−8.3 to −0.9)	0.017	0.80	Large
	End of pre‐swing (100%)	9.7 (7.2)	4.8 (5.3)	−4.9 (−8.7 to −1.1)	0.014	0.81	Large
	Statistical parametric mapping				0.044/0.002		
Knee joint – sagittal	Heel contact (0%)	10.7 (5.2)	12.1 (3.7)	1.4 (−1.2–4.0)	0.295	0.32	Small
	End of loading (20%)	19.4 (5.9)	23.8 (3.3)	4.4 (1.6–7.2)	0.003	0.94	Large
	End of midstance (50%)	13.1 (5.2)	15.1 (4.0)	2.0 (−0.7–4.7)	0.144	0.44	Small
	End of terminal stance (83%)	17.6 (4.4)	22.0 (4.2)	4.4 (1.9–6.9)	< 0.001	1.04	Large
	End of pre‐swing (100%)	48.5 (4.7)	52.1 (4.8)	3.6 (0.8–6.3)	0.013	0.77	Medium
	Statistical parametric mapping				0.021/0.026		
Ankle joint – sagittal	Heel contact (0%)	−7.0 (4.5)	−4.7 (3.5)	2.4 (0.0–4.7)	0.046	0.58	Medium
	End of loading (20%)	−9.3 (3.2)	−7.0 (3.6)	2.2 (0.2–4.2)	0.029	0.69	Medium
	End of midstance (50%)	0.3 (3.2)	2.8 (3.1)	2.5 (0.7–4.4)	0.008	0.81	Large
	End of terminal stance (83%)	7.0 (3.3)	8.4 (3.2)	1.4 (−0.5–3.3)	0.145	0.44	Small
	End of pre‐swing (100%)	−12.0 (7.1)	−15.5 (4.7)	−3.4 (−6.9 to −0.0)	0.054	0.59	Medium
	Statistical parametric mapping				0.002		
Subtalar joint	Heel contact (0%)	0.8 (3.7)	2.0 (3.1)	1.3 (−0.7–3.3)	0.210	0.36	Small
	End of loading (20%)	−5.8 (4.4)	−3.8 (3.1)	2.0 (−0.2–4.2)	0.081	0.54	Medium
	End of midstance (50%)	−4.4 (3.9)	−2.5 (3.4)	1.8 (−0.3–4.0)	0.091	0.53	Medium
	End of terminal stance (83%)	−0.8 (3.8)	2.3 (4.3)	3.1 (0.7–5.4)	0.011	0.78	Medium
	End of pre‐swing (100%)	1.5 (6.6)	1.5 (4.5)	−0.0 (−3.3 to 3.3)	0.982	0.00	Tiny
	Statistical parametric mapping				0.048		
Midtarsal joint	Heel contact (0%)	4.2 (4.6)	3.0 (2.2)	−1.1 (−3.3 to 1.0)	0.278	0.34	Small
	End of loading (20%)	1.2 (4.2)	−0.2 (2.4)	−1.4 (−3.4 to 0.6)	0.171	0.42	Small
	End of midstance (50%)	0.0 (4.3)	−1.3 (2.3)	−1.3 (−3.3 to 0.7)	0.191	0.39	Small
	End of terminal stance (83%)	1.1 (4.0)	0.2 (2.4)	−1.0 (−2.9 to 0.9)	0.311	0.30	Small
	End of pre‐swing (100%)	7.6 (4.3)	5.7 (2.7)	−2.0 (−4.1 to 0.1)	0.061	0.54	Medium
	Statistical parametric mapping				NS		
Tarsometatarsal joint	Heel contact (0%)	2.9 (6.5)	0.7 (2.2)	−2.2 (−5.1 to 0.6)	0.118	0.46	Medium
	End of loading (20%)	3.3 (5.7)	0.8 (1.7)	−2.5 (−5.0 to −0.0)	0.047	0.61	Medium
	End of midstance (50%)	3.0 (5.7)	0.7 (1.6)	−2.3 (−4.7 to 0.2)	0.069	0.56	Medium
	End of terminal stance (83%)	0.3 (5.5)	−1.2 (2.3)	−1.5 (−4.0 to 0.9)	0.211	0.36	Small
	End of pre‐swing (100%)	3.8 (6.9)	3.6 (4.2)	−0.1 (−3.5 to 3.2)	0.940	0.04	Tiny
	Statistical parametric mapping				NS		
Metatarsophalangeal joints	Heel contact (0%)	−23.5 (7.0)	−23.4 (6.7)	0.1 (−3.9–4.1)	0.960	0.01	Tiny
	End of loading (20%)	−16.1 (6.4)	−15.2 (6.7)	0.9 (−2.9–4.7)	0.646	0.14	Very small
	End of midstance (50%)	−13.6 (6.6)	−10.9 (5.7)	2.7 (−0.9–6.3)	0.139	0.45	Small
	End of terminal stance (83%)	−16.3 (6.6)	−13.5 (4.6)	2.8 (−0.5–6.1)	0.095	0.50	Small
	End of pre‐swing (100%)	−29.0 (7.4)	−31.3 (4.9)	−2.3 (−6.0 to 1.3)	0.203	0.37	Small
	Statistical parametric mapping				NS		

*Note:* Cohen's *d*. Interpretation: *<* 0.1 = tiny, 0.1 to *<* 0.2 = very small, 0.2 to *<* 0.5 = small, 0.5 to *<* 0.8 = medium, 0.8 to *<* 1.2 = large, 1.2–2 = very large, d > 2 = huge^39^. Values are mean (SD) unless otherwise indicated. For tri‐planar movements within the subtalar, midtarsal, and tarsometatarsal joints, positive values indicate supination, while negative values indicate pronation.

Abbreviation: NS: not significant.

For analysis using SPM, people with midfoot OA had less hip extension between 0% and 9% of stance, less hip extension between 30% and 77% of stance, less knee flexion and then less knee extension between 14% and 34% of stance, less knee flexion between 77% and 96% stance, less ankle dorsiflexion between 47% and 76% of stance, and greater subtalar pronation between 83% and 87% of stance. Significant findings are depicted as block shading in Figure 2 and the corresponding *p*‐values are in Table 2.

### 2.3 Stair Ascent and Descent

Of the 24 cases, 13 did not complete the stair ascent and were excluded due to balance concerns (*n* = 8) or insufficient kinematic data (*n* = 3). Additionally, one case was excluded from analysis because its matched control did not complete the stair ascent, resulting in 12 cases matched to 12 controls being analysed for this task. Similarly, some participants did not complete the stair descent and were excluded due to balance concerns (*n* = 10) or insufficient kinematic data (*n* = 2), leaving 12 cases for stair descent matched to 12 controls.

For stair ascent, there were no significant differences in ground contact time between people with midfoot OA (mean 1.36 s, SD 0.31) and controls (mean 1.28, SD 0.61; *p* = 0.71, *d* = 0.17: small effect). Similarly, for stair descent, there were no significant differences in ground contact time between people with midfoot OA (mean 1.09 s, SD 0.39) and controls (mean 1.00, SD 0.14; *p* = 0.46, *d* = 0.32: small effect). During stair ascent, people with midfoot OA had less hip extension at the end of loading and less MTP joint dorsiflexion at the end of terminal stance (see Table S1 and Figure S1). During stair descent, people with midfoot OA had less knee flexion at the end of pre‐swing (see Table S2 and Figure S2). There were no statistically significant differences between the groups in the SPM analyses for either stair ascent or descent.

## 3 Discussion

The objective of this study was to compare foot and lower limb kinematics between people with and without symptomatic radiographic midfoot OA when walking on level ground and when ascending and descending stairs. Our findings showed that during level walking, people with midfoot OA walked slower, had greater ground contact times and demonstrated differences in sagittal plane kinematics of the hip, knee and ankle. Additionally, there were differences in the kinematics of the subtalar and tarsometatarsal joints. The differences between the groups in stair ascent and descent were less pronounced, although the sample size for these comparisons was smaller due to the exclusion of some participants with balance concerns. These findings offer valuable insights into how people with midfoot OA walk and the mechanisms that may contribute to or result from the condition.

During level walking, those with midfoot OA walked slower, had greater contact times, and demonstrated different joint positions at the hip, knee, and ankle. Specifically, they showed less hip extension throughout stance, less knee flexion in early and late stance, and less ankle dorsiflexion throughout stance. In this study, we focussed solely on joint positions rather than overall joint motion. However, upon visually examining the curves in the kinematic figures without performing any statistical analysis, we observed that while the general joint movement patterns were relatively similar between the cases and controls, the joint positions differed. A possible explanation for the differences observed in joint positions in the hip, knee, and ankle is that, due to pain, people with midfoot OA may adopt a more cautious gait [10]. It is plausible that foot pain leads to reduced joint movement and increased stiffness as a protective response, serving as a pain avoidance mechanism to safeguard the affected foot [40].

Additionally, these differences could be related to walking speed. Since gait speed was self‐selected, participants with midfoot OA walked more slowly and had longer ground contact times, which may have contributed to reduced joint range of motion in the lower limb [41]. As a result, some of the observed differences may be influenced by variations in gait speed rather than midfoot OA itself. However, we intentionally allowed self‐selected speed to replicate participants' natural movement patterns in daily life. Both self‐selected and controlled walking speeds have advantages and disadvantages. After careful consideration, we opted against imposing a set speed, as this could lead to unnatural gait patterns. While self‐selected speed is a recognised limitation, it provides a more ecologically valid representation of how people with midfoot OA naturally move. Future research could explore controlled walking speeds to better isolate kinematic differences, but this must be balanced with ensuring participants' movements reflect real‐world function.

Similarly, people with midfoot OA have higher BMIs [23], which may influence gait mechanics. While a higher BMI has been linked to alterations in hip, knee, and ankle biomechanics, findings remain inconsistent across studies [42]. We chose not to control for BMI to better reflect the typical presentation of midfoot OA and capture the natural variation seen in those with the condition. Finally, although the predominance of women in this sample may limit generalisability to men, it reflects the higher prevalence of midfoot OA in women [23] and is therefore representative of the population most commonly affected.

A novel finding from this study was that those with midfoot OA had greater subtalar pronation in late stance. In our study, the subtalar, midtarsal and tarsometatarsal joint was modelled to measure the cardinal planes of motion (pronation and supination), which is a composite tri‐planar change in joint position. No previous studies have investigated the tri‐planar positions of the foot in this group, so it is difficult to compare with previous research. However, studies on static foot measures have found that people with midfoot OA tend to display a more pronated foot posture [9, 11]. Surrogate measures of dynamic motion, such as plantar pressures, have found that people with midfoot OA had pressures associated with lowering of the medial longitudinal arch, greater lateral push off and less propulsion at toe‐off [12].

During level walking, the subtalar joint typically starts in a supinated position at heel strike, pronates throughout the stance phase, and resupinates later in stance [43, 44]. In our study, the midfoot OA cases did not resupinate and remained in a more pronated position late in stance. It has been hypothesised that a potential cause of midfoot OA could be a more pronated foot posture, leading to increased joint compressive forces in the medial midfoot [9, 45]. This notion is supported by cadaver models, which demonstrate increased dorsal talonavicular joint compression when medial arch structures are sectioned to simulate a pronated foot [46, 47, 48]. As our study is cross‐sectional, we cannot rule out the possibility that changes in the subtalar joint occur after the onset and progression of midfoot OA. Nevertheless, our findings are significant, providing the first evidence of an association between greater subtalar pronation during late stance and midfoot OA It is worth noting that although previous static assessments of the foot have indicated a trend towards a more pronated foot posture [9, 11], kinematic studies have not found a strong correlation between static foot posture and kinematic measures [34, 49, 50]. Therefore, this finding should be regarded as a dynamic observation rather than a static pronated foot posture generalisation. Future prospective studies are needed to determine whether a more pronated subtalar joint plays a causal role in the development of midfoot OA.

We also found that midfoot OA cases displayed greater tarsometatarsal supination during early stance when analysed using the temporal events method [38], but not with SPM [18] analysis. The implication of this finding remains unclear. Although not statistically significant at all time points, there was an overall trend indicating that cases had a more pronated subtalar joint and a more supinated tarsometatarsal joint throughout most of stance. As the subtalar joint is in a more pronated position, we might expect the tarsometatarsal joint to be more supinated in order to maintain plantar contact with the ground [51, 52]. This is based on the twisted plate theory, which suggests that when the hindfoot and midfoot are pronated, the forefoot becomes supinated [51]. However, it is important to note that this model is theoretical, potentially limiting its relevance in contemporary contexts.

When climbing stairs, no significant differences in contact times were observed between midfoot OA cases and controls. However, stair climbing was associated with greater variability, as reflected by larger standard deviations, suggesting that participants adopted different strategies to complete the task. Stair ascent and descent were included in this study as they demand greater foot and ankle range of motion than level walking [53], potentially revealing deficits not evident during level ambulation. Notably, only around 60% of those with midfoot OA were able to complete the stair climbing trials, highlighting the challenges associated with this task, which may stem from issues with balance, strength, or pain. The minimal kinematic differences observed during stair ascent and descent may also reflect a selection bias, whereby those with better physical function and balance were more likely to complete the task. As a result, their movement patterns may have more closely resembled those of the control group, thereby reducing between‐group differences.

There are two kinematic studies on midfoot OA that have been reported [10, 13]. It is difficult to compare our findings with those of Deschamps et al. [10], as they investigated midfoot OA combined with subtalar joint OA. Additionally, they examined the three components of supination and pronation separately (dorsiflexion/plantarflexion, inversion/eversion, adduction/abduction) and focused on the total range of motion, whereas our study examined absolute joint positions. They did find that people with midfoot OA adopted a more cautious walking style, resulting in a pull‐off strategy rather than an active push‐off pattern [10]. Similarly, it is difficult to compare our study with that of Rao et al. [13], as they only investigated five foot joints and did not look at tri‐planar movements; they also focused on peak and total range of motion rather than absolute joint positions.

There are several strengths of this study. First, we used a validated lower limb model capable of analysing the tri‐planar movements of the foot joints. Second, cases were defined using a standardised foot manikin [24] to identify the location of foot pain and a validated atlas for documenting radiographic OA [22]. Third, the sample size is comparatively large compared to that of previous kinematic biomechanical studies in this area. This enhances the reliability and generalisability of the results, allowing for more precise estimates and a better representation of the broader population. Finally, SPM was employed alongside traditional methods for analysing gait data, providing a novel and more comprehensive approach that assesses gait across the entire gait cycle.

Nevertheless, several limitations warrant consideration. First, due to differing locations of midfoot OA, larger variability in gait patterns in the case group may have been observed. Second, climbing stairs inherently poses greater difficulty compared to overground walking, and this resulted in a smaller sample size due to many participants being unable to complete the task. Finally, as the study was cross‐sectional in design, we are unable to establish causal relationships.

## 4 Conclusions

People with midfoot OA walk slower, had longer ground contact times and demonstrate medium to large differences in sagittal plane hip, knee, and ankle kinematics, as well as medium differences in subtalar and tarsometatarsal kinematics. Differences between the groups for stair ascent and descent were less pronounced. Prospective studies are necessary to determine the temporal relationship between these findings and the development of midfoot OA.

## Author Contributions

**Merridy J. Lithgow:** conceptualisation, data Curation, formal analysis, investigation, methodology, project administration, software, visualisation, writing – original draft, writing – review and editing. **Jayishni N. Maharaj:** data curation, formal analysis, methodology, software, supervision, writing – review and editing. **Andrew K. Buldt:** conceptualisation, data curation, formal analysis, funding acquisition, investigation, methodology, supervision, writing – review and editing. **Shannon E. Munteanu:** conceptualisation, data curation, formal analysis, funding acquisition, methodology, supervision, writing – review and editing. **Benjamin F. Mentiplay:** formal analysis, writing – review and editing. **Hylton B. Menz:** conceptualisation, data curation, formal analysis, funding acquisition, methodology, supervision, visualisation, writing – review and editing.

## Conflicts of Interest

The authors declare no conflicts of interest.

## Supporting information

Supporting Information S1

Supporting Information S2

Figure S1

Figure S2

Table S1

Table S2

## Data Availability

The data that support the findings of this study are available from the corresponding author upon reasonable request.
