# Supplementary material for: Lower Limb Kinematics of People With Midfoot Osteoarthritis During Level Walking and Stair Climbing
Source: J Foot Ankle Res. 2025 Jun 9;18(2):e70054. doi: 10.1002/jfa2.70054 (PMC12146581; doi:10.1002/jfa2.70054)
Supplement: Supplementary file 1 — Supporting Information S1 [file JFA2-18-e70054-s005.docx]

**Supplementary file 1: Biomechanical data collection method for stair climbing**

For stair climbing, we assessed the ascent and descent phase using a two-step staircase (step height 180 mm and step depth of 300 mm). During stair ascent, participants were instructed to take one step up to the first level with their index foot, and then to place their contralateral leg on the second step. Stance phase of the index foot (first step) was analysed. For stair descent, this process was reversed so that the participant's first step with their index foot was analysed. As there were no rails for support, data collection did not proceed if participants were unable to ascend or descend without support due to safety and balance concerns. After familiarisation, participants were instructed to ascend and descend the staircase until they completed a minimum of three successful trials of data collection, with each trial's step ground contact time falling within 15% of the participant's average.
