# Supplementary material for: Lower Limb Kinematics of People With Midfoot Osteoarthritis During Level Walking and Stair Climbing
Source: J Foot Ankle Res. 2025 Jun 9;18(2):e70054. doi: 10.1002/jfa2.70054 (PMC12146581; doi:10.1002/jfa2.70054)
Supplement: Supplementary file 2 — Supporting Information S2 [file JFA2-18-e70054-s003.docx]

**Supplementary file 2: Musculoskeletal modelling**

A validated multi-segment foot model embedded in a lower limb model was implemented in OpenSim software^1^ for kinematic analysis. The model is capable of measuring motion across five joints: the subtalar joint (connecting the talus and calcaneus), the midtarsal joint (connecting the calcaneus and midfoot), the tarsometatarsal joint (connecting the midfoot and forefoot), and the metatarsophalangeal joint (connecting the forefoot and toes). The model used in this study uses inverse kinematics approach that assumes fixed degrees of freedom at each joint^2^. The dimensions and masses of each segment were scaled using static calibration markers on the segment and body mass respectively^2^. Briefly, the four joints that connect the five foot segments are as follows: the subtalar joint (connecting the talus and calcaneus), the midtarsal joint (connecting the calcaneus and midfoot), the tarsometatarsal joint (connecting the midfoot and forefoot), and the metatarsophalangeal joint (connecting the forefoot and toes). The subtalar joint, midtarsal joint, and tarsometatarsal joint were modelled as revolute joints. For each joint, an inclination and deviation angle were defined to quantify the orientation of the joint axes. The inclination angle is the angle between the sagittal projection of the axis and the horizontal plane, while the deviation angle is measured along the horizontal projection with respect to the long axis of the foot^3^. Specifically, the joint axis of the subtalar joint has a 37° inclination and a 21° deviation to the midline of the body, running from posterior, inferior, and lateral to anterior, superior, and medial through the rearfoot^4^. The midtarsal joint axis is oriented in an upward, medial direction with a 37.9° inclination and a 29° deviation to the midline of the body^5^. The tarsometatarsal joint axis has an inclination of 10° and a deviation of -70° from the midline of the body, running in a medial, upward direction from the fifth to the second metatarsal. The modelled oblique axis allows these joints to perform triplanar motion of pronation and supination^6^.

The model was first scaled to the dimensions of each participant using a static calibration trial. In the static trial participants stood in a comfortable position whilst the positions of all markers were recorded. Marker positions in the static trial were then used to scale the generic model segments to the dimensions of the participants to create a scaled model of each participant. This scaling was based on pairs of calibration markers on each segment. A scale factor for each segment was calculated by dividing the distance between the two calibration markers of the segment on the participant by the distance between the same markers on the generic model. The scale factor was subsequently multiplied by the dimensions of the segment in the generic model to create a segment scaled specifically to the participant. Segment masses were scaled to sum to the mass of the participant's lower body (46% total body mass) and to keep the distribution of mass among segments the same as is in the generic model. The inertia tensor describing each segment's inertial properties was then updated for the new dimensions and mass. The pairs of markers for each segment were: pelvis - right and left anterior-superior iliac spines and right and left posterior-superior iliac spines; thigh - anterior-superior iliac spine and lateral knee joint; shank - lateral knee joint and lateral malleolus; rearfoot (width) – medial and lateral calcaneus marker; rearfoot (depth) – distal calcaneus and metatarsal base of digit one; rearfoot (width) – medial and lateral calcaneus; mid- and fore- foot (width) – metatarsal head of digit one and digit five. Mid- and fore- foot (height) – lateral malleolus marker and metatarsal head of digit one; mid- and fore- foot (depth) – base and head of the fifth metatarsal.

**Table 1.** Description of the body segment marker location^2, 7^

| Segment | Name | Description |
| --- | --- | --- |
| Hip | ASIS  PSIS | Marker placed on the anterior superior iliac spine  Marker placed on the posterior superior iliac spine |
| Thigh  Knee  Shank  Ankle  Rearfoot  Midfoot  Forefoot  Digits | T1-4  MKN  LKN  S1-4  MM  LM  PC  DC  MC  LC  NAV  MB1  MB2  MB5  MH1  MH2  MH5  D1  D2  D5 | Cluster placed on lateral aspect of the lower third of the thigh  Marker placed on medial epicondyle of the knee  Marker placed on lateral epicondyle of the knee  Cluster placed on lateral aspect of leg  Marker placed on medial malleolus  Marker placed on lateral malleolus  Marker placed on posterior calcaneus (superior)  Marker placed on posterior calcaneus (inferior)  Marker placed on sustentaculum tali  Marker placed on peroneal tubercle  Marker placed on navicular tuberosity  Marker placed on base of the first metatarsal  Marker placed on base of the second metatarsal  Marker placed on base of the fifth metatarsal  Marker placed on head of the first metatarsal  Marker placed on head of the second metatarsal  Marker placed on head of the fifth metatarsal  Marker placed on the proximal phalanx of the hallux  Marker placed on the proximal phalanx of second digit  Marker placed on the proximal phalanx of fifth digit |

**
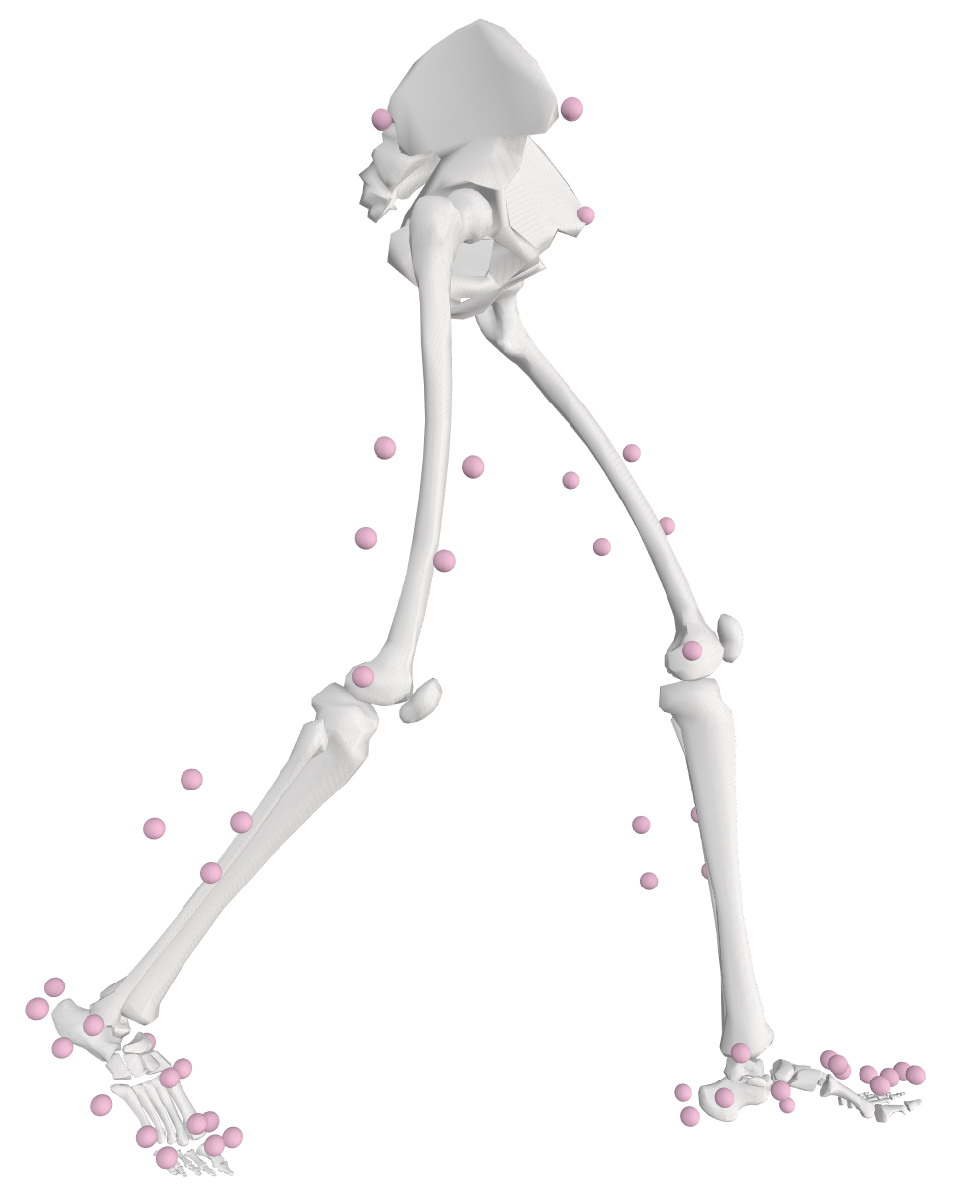

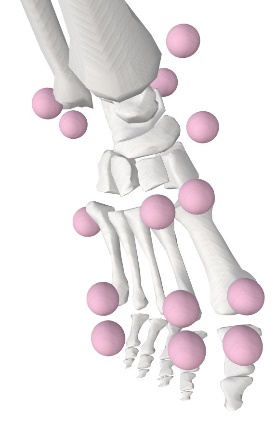
**

**REFERENCES**

1. Delp SL, Anderson FC, Arnold AS, Loan P, Habib A, John CT, et al. OpenSim: open-source software to create and analyze dynamic simulations of movement. IEEE Trans Biomed Eng 2007; 54: 1940-1950.

2. Maharaj JN, Cresswell AG, Lichtwark GA. Foot structure is significantly associated to subtalar joint kinetics and mechanical energetics. Gait Posture 2017; 58: 159-165.

3. van den Bogert AJ, Smith GD, Nigg BM. In vivo determination of the anatomical axes of the ankle joint complex: an optimization approach. J Biomech 1994; 27: 1477-1488.

4. Lewis GS, Kirby KA, Piazza SJ. Determination of subtalar joint axis location by restriction of talocrural joint motion. Gait Posture 2007; 25: 63-69.

5. Nester CJ, Findlow A, Bowker P. Scientific approach to the axis of rotation at the midtarsal joint. J Am Podiatr Med Assoc 2001; 91: 68-73.

6. Maharaj JN, Cresswell AG, Lichtwark GA. The Immediate Effect of Foot Orthoses on Subtalar Joint Mechanics and Energetics. Med Sci Sports Exerc 2018; 50: 1449-1456.

7. Maharaj JN, Rainbow MJ, Cresswell AG, Kessler S, Konow N, Gehring D, et al. Modelling the complexity of the foot and ankle during human locomotion: the development and validation of a multi-segment foot model using biplanar videoradiography. Comput Methods Biomech Biomed Engin 2022; 25: 554-565.
