# Supplementary material for: Lower Limb Kinematics of People With Midfoot Osteoarthritis During Level Walking and Stair Climbing
Source: J Foot Ankle Res. 2025 Jun 9;18(2):e70054. doi: 10.1002/jfa2.70054 (PMC12146581; doi:10.1002/jfa2.70054)
Supplement: Supplementary file 3 — Figure S1 [file JFA2-18-e70054-s002.docx]

**Supplementary Figure 1:** Absolute angles of lower limb joint kinematics during stair ascent in people with symptomatic midfoot OA and asymptomatic controls (mean and standard error bars). The black line represents the cases, and the grey line represents the controls. The horizontal axis shows the percentage of the stance phase, while the vertical axis indicates degrees of movement. Significant findings using the temporal events method are marked with vertical lines, with one asterisk representing *p*<0.05 and two asterisks representing *p*<0.01. Significant findings from the SPM analysis are highlighted with block shading.

**
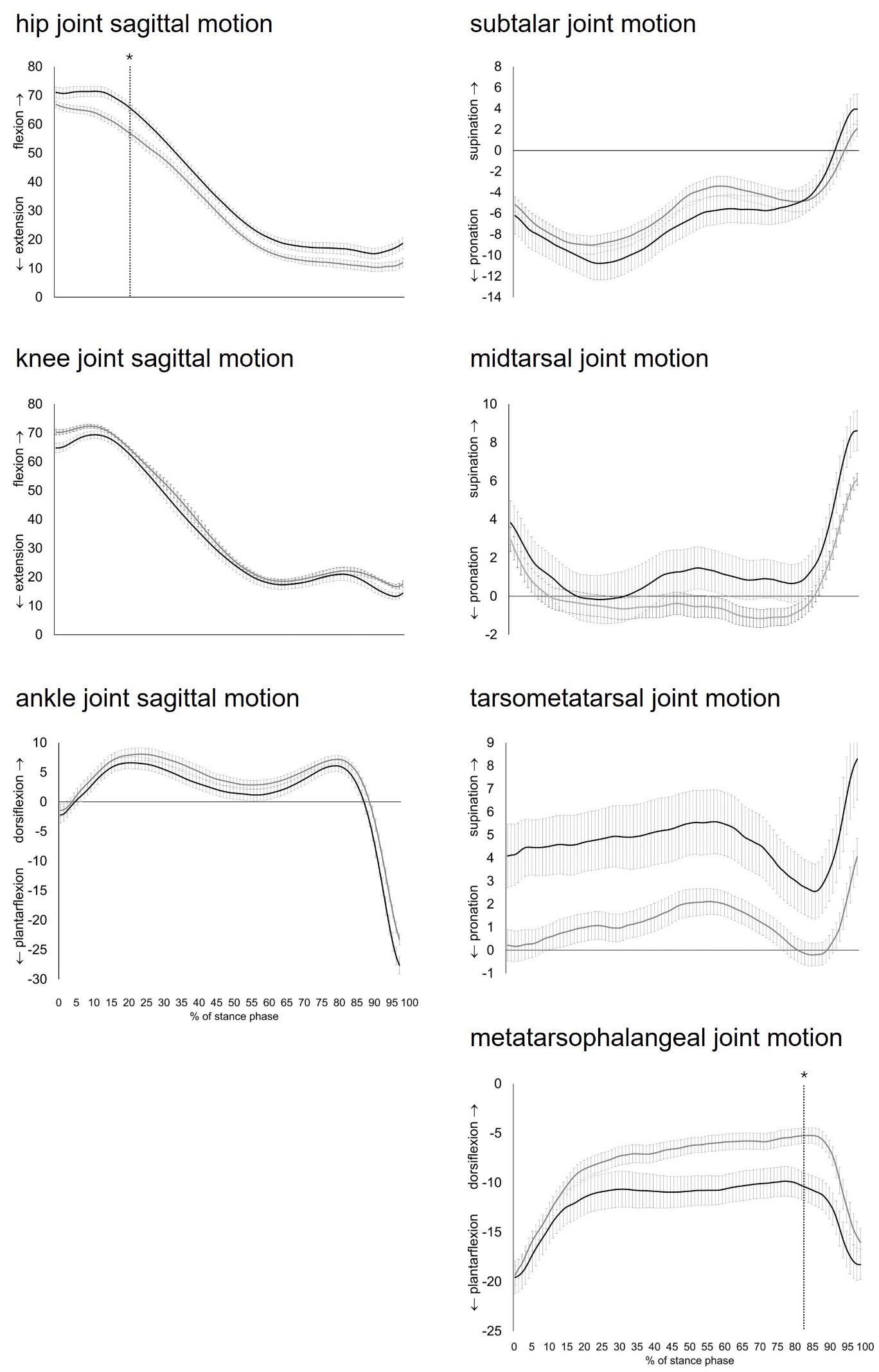
**
