# Supplementary material for: Lower Limb Kinematics of People With Midfoot Osteoarthritis During Level Walking and Stair Climbing
Source: J Foot Ankle Res. 2025 Jun 9;18(2):e70054. doi: 10.1002/jfa2.70054 (PMC12146581; doi:10.1002/jfa2.70054)
Supplement: Supplementary file 5 — Table S1 [file JFA2-18-e70054-s001.docx]

**Supplementary Table 1:** Absolute angles of lower limb joint kinematics during stair ascent in people with symptomatic midfoot OA and asymptomatic controls. Values are mean (SD) unless otherwise indicated. For tri-planar movements within the subtalar, midtarsal, and tarsometatarsal joints, positive values indicate supination, while negative values indicate pronation.

|  |  | cases (n=12) | controls (n=12) | mean difference (95% CI) | *p*-value | effect size (Cohen’s *d*) | interpretation |
| --- | --- | --- | --- | --- | --- | --- | --- |
| hip joint – sagittal | foot contact (0%) | 71.1 (9.3) | 66.9 (5.5) | -4.2 (-10.8 to 2.3) | 0.193 | 0.57 | medium |
|  | end of loading (20%) | 66.9 (8.0) | 58.0 (8.3) | -8.9 (-15.8 to -2.0) | 0.014 | 1.14 | large |
|  | end of midstance (50%) | 30.5 (6.7) | 25.3 (6.5) | -5.2 (-10.8 to 0.4) | 0.066 | 0.82 | large |
|  | end of terminal stance (83%) | 16.8 (9.2) | 11.5 (7.3) | -5.3 (-12.4 to 1.7) | 0.129 | 0.67 | medium |
|  | end of pre-swing (100%) | 18.6 (9.7) | 11.9 (7.7) | -6.7 (-14.1 to 0.8) | 0.076 | 0.80 | large |
|  | statistical parametric mapping |  |  |  | NS |  |  |
| knee joint – sagittal | foot contact (0%) | 64.8 (8.2) | 70.3 (4.5) | 5.5 (-0.2 to 11.2) | 0.53 | 0.87 | large |
|  | end of loading (20%) | 64.0 (9.7) | 65.9 (2.6) | 1.9 (-4.4 to 8.2) | 0.525 | 0.28 | small |
|  | end of midstance (50%) | 25.6 (11.1) | 27.2 (4.6) | 1.6 (-5.8 to 9.0) | 0.652 | 0.20 | small |
|  | end of terminal stance (83%) | 21.0 (10.8) | 22.1 (6.5) | 1.2 (-6.4 to 8.7) | 0.754 | 0.13 | very small |
|  | end of pre-swing (100%) | 14.4 (5.6) | 17.6 (5.5) | 3.2 (-1.5 to 7.8) | 0.174 | 0.60 | medium |
|  | statistical parametric mapping |  |  |  | NS |  |  |
| ankle joint – sagittal | foot contact (0%) | -2.2 (6.6) | -1.4 (5.2) | 0.8 (-4.2 to 5.8) | 0.748 | 0.14 | very small |
|  | end of loading (20%) | 6.6 (4.9) | 7.9 (5.3) | 1.3 (-3.0 to 5.6) | 0.543 | 0.27 | small |
|  | end of midstance (50%) | 1.6 (5.2) | 3.3 (4.1) | 1.8 (-2.2 to 5.7) | 0.371 | 0.38 | small |
|  | end of terminal stance (83%) | 5.9 (4.9) | 7.1 (3.1) | 1.2 (-2.3 to 4.7) | 0.473 | 0.31 | small |
|  | end of pre-swing (100%) | -27.6 (7.1) | -23.3 (4.6) | 4.4 (-0.7 to 9.5) | 0.088 | 0.75 | medium |
|  | statistical parametric mapping |  |  |  | NS |  |  |
| subtalar joint | foot contact (0%) | -6.2 (8.8) | -5.2 (3.3) | 1.0 (-4.8 to 6.8) | 0.707 | 0.16 | very small |
|  | end of loading (20%) | -10.3 (7.9) | -9.0 (4.3) | 1.3 (-4.1 to 6.8) | 0.613 | 0.21 | small |
|  | end of midstance (50%) | -6.8 (6.7) | -4.4 (4.0) | 2.4 (-2.3 to 7.1) | 0.309 | 0.45 | small |
|  | end of terminal stance (83%) | -5.0 (6.7) | -4.9 (4.8) | 0.1 (-4.8 to 5.0) | 0.967 | 0.02 | tiny |
|  | end of pre-swing (100%) | 3.9 (7.1) | 2.1 (3.7) | -1.9 (-6.6 to 2.9) | 0.426 | 0.33 | small |
|  | statistical parametric mapping |  |  |  | NS |  |  |
| midtarsal joint | foot contact (0%) | 3.8 (5.5) | 3.0 (3.0) | -0.9 (-4.7 to 2.9) | 0.636 | 0.19 | very small |
|  | end of loading (20%) | -0.0 (5.9) | -0.4 (2.8) | -0.3 (-4.3 to 3.7) | 0.869 | 0.09 | tiny |
|  | end of midstance (50%) | 1.3 (5.4) | -0.4 (2.8) | -1.7 (-5.5 to 2.0) | 0.339 | 0.41 | small |
|  | end of terminal stance (83%) | 0.7 (4.8) | -0.8 (2.1) | -1.5 (-4.7 to 1.7) | 0.329 | 0.42 | small |
|  | end of pre-swing (100%) | 8.6 (5.1) | 6.1 (1.5) | -2.5 (-5.9 to 0.8) | 0.127 | 0.69 | medium |
|  | statistical parametric mapping |  |  |  | NS |  |  |
| tarsometatarsal joint | foot contact (0%) | 4.1 (6.7) | 0.2 (3.3) | -3.9 (-8.5 to 0.7) | 0.094 | 0.77 | medium |
|  | end of loading (20%) | 4.6 (6.3) | 0.9 (3.0) | -3.7 (-7.9 to 0.6) | 0.088 | 0.78 | medium |
|  | end of midstance (50%) | 5.4 (6.8) | 2.0 (3.0) | -3.4 (-8.0 to 1.1) | 0.131 | 0.68 | medium |
|  | end of terminal stance (83%) | 2.9 (6.0) | 0.0 (2.5) | -2.9 (-6.9 to 1.1) | 0.138 | 0.66 | medium |
|  | end of pre-swing (100%) | 8.3 (8.7) | 4.1 (3.9) | -4.2 (-10.1 to 1.6) | 0.145 | 0.65 | medium |
|  | statistical parametric mapping |  |  |  | NS |  |  |
| metatarsophalangeal joints | foot contact (0%) | -19.6 (8.0) | -19.3 (4.8) | 2.7 (-5.4 to 5.9) | 0.936 | 0.05 | tiny |
|  | end of loading (20%) | -11.5 (8.5) | -8.6 (4.4) | 2.9 (-3.0 to 8.7) | 0.319 | 0.45 | small |
|  | end of midstance (50%) | -10.9 (7.5) | -6.3 (3.8) | 4.6 (-0.6 to 9.7) | 0.077 | 0.81 | large |
|  | end of terminal stance (83%) | -10.3 (7.0) | -5.3 (3.8) | 5.0 (0.3 to 9.8) | 0.039 | 0.93 | large |
|  | end of pre-swing (100%) | -18.3 (7.6) | -16.0 (7.1) | 2.2 (-4.0 to 8.5) | 0.466 | 0.33 | small |
|  | statistical parametric mapping |  |  |  | NS |  |  |

Cohen’s *d*. Interpretation: *<* 0.1 = tiny, 0.1 to *<* 0.2 = very small, 0.2 to *<* 0.5 = small, 0.5 to *<* 0.8 = medium, 0.8 to *<* 1.2 = large, 1.2–2 - very large, d *>* 2 = huge^39^. NS: not significant.
