# Supplementary material for: Lower Limb Kinematics of People With Midfoot Osteoarthritis During Level Walking and Stair Climbing
Source: J Foot Ankle Res. 2025 Jun 9;18(2):e70054. doi: 10.1002/jfa2.70054 (PMC12146581; doi:10.1002/jfa2.70054)
Supplement: Supplementary file 6 — Table S2 [file JFA2-18-e70054-s004.docx]

**Supplementary Table 2:** Absolute angles of lower limb joint kinematics during stair descent in people with symptomatic midfoot OA and asymptomatic controls. Values are mean (SD) unless otherwise indicated. For tri-planar movements within the subtalar, midtarsal, and tarsometatarsal joints, positive values indicate supination, while negative values indicate pronation.

|  |  | cases (n=12) | controls (n=12) | mean difference (95% CI) | *p*-value | effect size | interpretation |
| --- | --- | --- | --- | --- | --- | --- | --- |
| hip joint – sagittal | foot contact (0%) | 25.6 (7.4) | 22.4 (7.1) | -3.2 (-9.3 to 2.9) | 0.292 | 0.46 | small |
|  | end of loading (20%) | 26.1 (8.7) | 24.8 (8.4) | -1.3 (-8.6 to 5.9) | 0.710 | 0.16 | very small |
|  | end of midstance (50%) | 21.5 (8.0) | 16.0 (9.3) | -5.5 (-12.8 to 1.8) | 0.134 | 0.66 | medium |
|  | end of terminal stance (83%) | 32.6 (11.9) | 26.9 (13.5) | -5.7 (-16.5 to 5.1) | 0.286 | 0.47 | small |
|  | end of pre-swing (100%) | 42.1 (12.9) | 36.6 (10.6) | -5.5 (-15.5 to 4.5) | 0.263 | 0.49 | small |
|  | statistical parametric mapping |  |  |  | NS |  |  |
| knee joint – sagittal | foot contact (0%) | 9.4 (7.1) | 13.4 (4.4) | 3.9 (-1.1 to 9.0) | 0.120 | 0.71 | medium |
|  | end of loading (20%) | 26.2 (11.6) | 33.6 (6.9) | 3.9 (-0.7 to 15.4) | 0.071 | 0.81 | large |
|  | end of midstance (50%) | 32.5 (12.6) | 31.2 (4.6) | -1.3 (-9.6 to 7.0) | 0.739 | 0.14 | very small |
|  | end of terminal stance (83%) | 75.8 (18.1) | 75.1 (12.5) | -0.6 (-13.8 to 12.5) | 0.921 | 0.05 | tiny |
|  | end of pre-swing (100%) | 100.7 (6.6) | 105.8 (3.5) | 5.1 (0.5 to 9.6) | 0.031 | 1.01 | large |
|  | statistical parametric mapping |  |  |  | NS |  |  |
| ankle joint – sagittal | foot contact (0%) | -28.3 (5.7) | -30.0 (2.9) | -1.7 (-5.6 to 2.2) | 0.361 | 0.39 | small |
|  | end of loading (20%) | 0.8 (6.5) | 4.0 (4.4) | 3.3 (-1.4 to 7.9) | 0.163 | 0.6 | medium |
|  | end of midstance (50%) | 9.6 (8.0) | 9.1 (4.2) | -0.4 (-5.9 to 5.0) | 0.866 | 0.08 | tiny |
|  | end of terminal stance (83%) | 19.4 (10.2) | 19.1 (3.9) | -0.3 (-7.0 to 6.4) | 0.922 | 0.04 | tiny |
|  | end of pre-swing (100%) | 3.9 (8.8) | 1.9 (7.0) | -1.9 (-8.7 to 4.8) | 0.556 | 0.26 | small |
|  | statistical parametric mapping |  |  |  | NS |  |  |
| subtalar joint | foot contact (0%) | 0.78 (4.1) | 0.56 (3.5) | -0.2 (-3.4 to 3.0) | 0.891 | 0.06 | tiny |
|  | end of loading (20%) | -5.3 (5.4) | -5.9 (3.6) | -0.6 (-4.5 to 3.2) | 0.735 | 0.14 | very small |
|  | end of midstance (50%) | -7.1 (6.2) | -6.5 (3.6) | 0.6 (-3.7 to 4.9) | 0.774 | 0.12 | very small |
|  | end of terminal stance (83%) | -0.4 (10.2) | -4.8 (5.0) | -4.4 (-11.2 to 2.5) | 0.198 | 0.57 | medium |
|  | end of pre-swing (100%) | -0.6 (8.8) | -4.0 (5.3) | -4.6 (-10.8 to 1.5) | 0.133 | 0.49 | small |
|  | statistical parametric mapping |  |  |  | NS |  |  |
| midtarsal joint | foot contact (0%) | 7.4 (4.5) | 4.4 (2.5) | -3.0 (-6.0 to 0.1) | 0.054 | 0.86 | large |
|  | end of loading (20%) | 1.0 (4.2) | -0.9 (3.0) | -1.9 (-5.0 to 1.2) | 0.215 | 0.54 | medium |
|  | end of midstance (50%) | -0.5 (4.2) | -1.2 (2.8) | -0.7 (-3.8 to 2.3) | 0.615 | 0.2 | small |
|  | end of terminal stance (83%) | 4.3 (4.9) | 3.7 (2.6) | -0.6 (-4.0 to 2.7) | 0.697 | 0.16 | very small |
|  | end of pre-swing (100%) | 7.0 (5.2) | 6.4 (3.8) | -0.6 (-4.5 to 3.3) | 0.753 | 0.14 | very small |
|  | statistical parametric mapping |  |  |  | NS |  |  |
| tarsometatarsal joint | foot contact (0%) | 6.5 (7.6) | 4.2 (2.3) | -2.3 (-7.2 to 2.7) | 0.343 | 0.43 | small |
|  | end of loading (20%) | 1.8 (7.2) | -2.3 (1.7) | -4.1 (-8.7 to 0.4) | 0.081 | 0.82 | large |
|  | end of midstance (50%) | 1.8 (6.6) | -2.5 (1.8) | -4.3 (-8.5 to 0.0) | 0.050 | 0.93 | large |
|  | end of terminal stance (83%) | 1.6 (8.5) | -3.5 (3.5) | -5.1 (-10.7 to 0.6) | 0.075 | 0.82 | large |
|  | end of pre-swing (100%) | 5.0 (8.6) | 2.3 (5.5) | -2.6 (-8.8 to 3.5) | 0.381 | 0.39 | small |
|  | statistical parametric mapping |  |  |  | NS |  |  |
| metatarsophalangeal joints | foot contact (0%) | -13.8 (7.8) | -13.2 (4.6) | 0.7 (-4.8 to 6.1) | 0.805 | 0.1 | very small |
|  | end of loading (20%) | -10.3 (6.9) | -8.2 (4.7) | 2.1 (-2.9 to 7.1) | 0.394 | 0.37 | small |
|  | end of midstance (50%) | -10.0 (7.9) | -6.5 (4.6) | 3.5 (-2.0 to 9.1) | 0.199 | 0.57 | medium |
|  | end of terminal stance (83%) | -20.1 (13.0) | -15.9 (6.8) | 4.2 (-4.6 to 13.0) | 0.332 | 0.42 | small |
|  | end of pre-swing (100%) | -27.3 (10.5) | -30.7 (4.4) | -3.4 (-10.4 to 3.6) | 0.317 | 0.44 | small |
|  | statistical parametric mapping |  |  |  | NS |  |  |

Cohen’s *d*. Interpretation: *<* 0.1 = tiny, 0.1 to *<* 0.2 = very small, 0.2 to *<* 0.5 = small, 0.5 to *<* 0.8 = medium, 0.8 to *<* 1.2 = large, 1.2–2 - very large, d *>* 2 = huge^39^. NS: not significant.
